# Racism and alterations in brain structure and function: A narrative review of emerging neuroimaging evidence in racialized individuals

**DOI:** 10.3758/s13415-026-01461-x

**Published:** 2026-06-09

**Authors:** Seyed Mohammad Mahdi Moshirian Farahi, Jude Mary Cénat

**Affiliations:** 1 School of Psychology, Faculty of Social Sciences, University of Ottawa, Ottawa, ON Canada; 2 Interdisciplinary Centre for Black Health, University of Ottawa, Ottawa, Ontario Canada; 3 University of Ottawa Research Chair on Black Health, Ottawa, Ontario Canada

**Keywords:** Racial discrimination, Racism, Neuroimaging, Magnetic resonance imaging, Brain structure and function, Brain alterations, Racialized individuals

## Abstract

Racial discrimination functions as a chronic psychosocial stressor that underlies racial trauma and elevates risk for mental disorders among racialized individuals. Emerging neuroimaging research has identified associations between experiences of racial discrimination and alterations in brain structure and function. However, the limited number of studies among racialized individuals constitutes a major gap in the scientific literature, leading to critical deficiencies in scientific knowledge, clinical practice, and informed public health strategies. This narrative review synthesizes findings from 19 neuroimaging studies examining the associations between experiences of racial discrimination and brain structure and function among racialized individuals across the lifespan. Neuroimaging studies have revealed consistent alterations in neural circuits involved in emotion regulation, threat detection, cognitive control, and self-referential processing among Black and other racialized individuals with experiences of racial discrimination. Notably, experiences of racial discrimination are associated with increased salience network connectivity (e.g., amygdala-insula), reduced prefrontal connectivity, decreased white matter integrity, and cortical thinning in regions, such as the cingulate cortex and hippocampus. These neural alterations are linked to heightened vigilance, emotion dysregulation, and higher risk for mental and physical health problems. This review highlights the limitations of previous studies along with providing future research directions. Recent neuroimaging studies on racial discrimination reveal key methodological limitations, including reliance on cross-sectional designs, limited sample diversity, small sample sizes, and inadequate intersectional analyses. Future research should employ longitudinal, multimodal approaches with larger, more diverse samples to elucidate causal pathways and protective factors underlying racism-related neurobiological outcomes.

**Supplementary Information:**

The online version contains supplementary material available at https://doi.org/10.3758/s13415-026-01461-x.

## Introduction

Racism is a system of beliefs, social practices, institutional policies, and structural mechanisms that privilege individuals positioned at the top of the racial hierarchy while disadvantaging racialized groups (Bonilla-Silva, 1997; Clark et al., 1999; Haeny et al., 2021). One key manifestation of racism is racial discrimination (Cénat, 2023; Williams & Mohammed, 2009). Racial discrimination involves actions and practices by dominant groups that negatively and disproportionately impact ethnic groups (Seaton, 2006). Racial discrimination can manifest explicitly or implicitly and can occur at both institutional and structural levels (Banaji et al., 2021; Braveman et al., 2022; Haeny et al., 2021; Matthew, 2024; National Research Council et al., 2004). Racial discrimination includes racial bias or stereotypes that might affect decision-making processes in different contexts, such as education, healthcare system, policies, housing, criminal justice, and employment (Cénat et al., 2022; Gopal et al., 2021; Williams & Mohammed, 2009).

Previous studies have shown a high prevalence of racial discrimination among racialized individuals in Western countries, including the US, Canada, and United Kingdom. Racialized individuals are those who are considered part of a minority ethno-racial group and subjected to discrimination given their race (Immigration, Refugees and Citizenship Canada, 2022). For instance, Lee et al. (2019) reported a prevalence of racial discrimination ranging from 50% to 75% among racialized groups (e.g., Black, Hispanic, and Asian individuals) in the US. Studies have shown higher racial discrimination among Black (Cotter, 2022; Lee et al., 2019) and Indigenous individuals (Cotter, 2022) compared to nonracialized individuals (i.e., White, non-Indigenous, nonvisible minority population). The impact of racial discrimination on mental and physical health is profound, leading to higher rates of stress, anxiety, depression, and chronic illnesses among racialized individuals who have experienced racial discrimination (Agbonlahor et al., 2024; Burgess et al., 2009; Busse et al., 2017; Calvin et al., 2003; Cénat et al., 2022; Cénat et al., 2025a, b; Chae et al., 2012; Hwang & Goto, 2008; Kogan et al., 2022; Njoroge et al., 2021; Paradies, 2018; Shepherd et al., 2017; Siddiqi et al., 2017; Simons et al., 2018, 2021; Vines et al., 2017). Most existing efforts in racism research have concentrated on sociocultural aspects, while neurobiological mechanisms of racism remain understudied. Understanding the brain network and functions is crucial, because racial discrimination may lead to long-term mental health issues (Assari et al., 2017; Cave et al., 2020; Cénat, 2023; Guerrero et al., 2023; Kwate & Goodman, 2015).

It is very important to understand how racial discrimination affects brain structure and function in racialized individuals as repetitive exposure to racism might lead to racial trauma and mental health issues (Cénat, 2023; Comas-Díaz et al., 2019; Saleem et al., 2020) and influence neural circuits involved in emotion regulation, threat detection, and cognitive control (Cole et al., 2024; Fani et al., 2021; Grasser & Jovanovic, 2022; Keating et al., 2022). According to the complex racial trauma (CoRT) model, repeated and chronic experiences of racial discrimination across interconnected levels (e.g., interpersonal, institutional, and structural) initiate a cumulative stress process that, over time, develops into racial trauma (Cénat, 2023). Within this framework, racial trauma is conceptualized as a multidimensional and systemic condition that becomes embedded in neurobiological functioning, shapes cognitive and emotional processes, disrupts social relationships, and contributes to enduring psychological, interpersonal, socioeconomic, and biological consequences. It is termed complex racial trauma, as it affects multiple spheres of an individual’s life, produces enduring consequences across the lifespan, and may involve intergenerational and epigenetic impacts (Cénat, 2023). Hence, consistent with the theorization of complex racial trauma, the impacts of racial discrimination must be examined from a developmental and longitudinal perspective, with explicit attention to their cumulative and lifespan trajectories, intergenerational transmission, and neurobiological sequelae, including alterations in brain structure and function. The neural pathways underlying these mechanisms, including changes in cognition, mood, physiological arousal and reactivity, hypervigilance, and neurodevelopment, should be investigated. Given the chronic and cumulative nature of racial discrimination as a stressor (Moshirian Farahi & Cénat, 2026), it is expected that repeated racial discrimination activates stress-response systems (e.g., hypothalamic-pituitary-adrenal axis [HPA]; Elbasheir et al., 2024c), leading over time to neurobiological embedding that alters brain networks. In this line, Muscatell et al. (2022) hypothesize that racial discrimination is associated with three core brain networks, including affective processing (e.g., salience network), memory (e.g., default mode network), and cognitive control (e.g., executive control network). These neural alterations are expected to partially overlap with stress- and trauma-related neural patterns observed in conditions, such as posttraumatic stress disorder (PTSD), and linked to hypervigilance (e.g., amygdala) and altered network connectivity (Hobson et al., 2022).

Racialized individuals remain underrepresented in neuroimaging research, leading to gaps in scientific knowledge that can arise systemic bias in diagnosis, treatment, and intervention strategies. Racialized individuals are often underrepresented in neuroscience research for several reasons, such as research-related, systemic, and individual factors. For example, studies may rely on more homogeneous samples in the neuroimaging and experimental studies, limiting generalizability, and the infrequent reporting of racial demographics (Goldfarb & Brown, 2022; Harnett et al., 2025). The other reason can be due to systemic barriers, such as inequitable neuroimaging devices. For instance, EEG research is particularly limited among Black individuals (Choy et al., 2022). Limited funding further restricts researchers from conducting neuroimaging studies, given their high costs (Harnett et al., 2025). It can also be linked to participants’ willingness and ongoing mistrust to research (Milani et al., 2021), which require building trust between researchers and community members (Harnett et al., 2025). This review paper highlights the importance of racial discrimination as a race-based stressor in brain activity and highlights current research gaps and outlines future directions in the field. The narrative review synthesizes findings from 19 studies that have focused on the relation between racial discrimination and the brain in racialized individuals. These studies have employed various neuroimaging techniques, including resting-state functional magnetic resonance imaging (fMRI), structural MRI, and task-based fMRI, to explore the associations between racial discrimination and brain structure/function among Black/African American populations.

## Methods

A narrative review was conducted to identify peer-reviewed papers written in English that have concentrated on racial discrimination and the brain in racialized individuals, “racial discrimination,” “brain,” “fMRI,” and “MRI” keywords were used primarily in the PsycInfo database, with supplementary searches in PubMed to ensure comprehensive coverage of relevant studies. These terms were selected to capture relevant neuroimaging research focused on racial discrimination in racialized individuals. No restrictions were applied based on year of publication (any date), country, or population subgroup. Studies were considered eligible if they (a) explicitly measured racial discrimination or racism (e.g., self-reported experiences of racial discrimination), (b) included neuroimaging (structural MRI, resting-state fMRI, or task-based fMRI), and (c) focused on racialized populations. Nonempirical articles, review articles, qualitative studies, and studies without neuroimaging data were excluded. The search was conducted in May 2025.

Initial database results yielded 232 records, which most studies did not include neuroimaging data, and these were subsequently screened at the title and abstract level for relevance. Full text papers were then reviewed to confirm eligibility based on the criteria provided above. Screening was conducted by the first author. Any uncertainties regarding study eligibility were resolved through consultation with a second author. Based on our search, most studies were published since 2020, and earlier studies (Masten et al., 2011) were retained for their conceptual relevance. No relevant studies were found prior to 2011. Totally, 19 eligible peer-reviewed articles were identified. In general, these studies have utilized a range of neuroimaging methods, such as resting-state fMRI, structural MRI, and task-based fMRI. Key characteristics and findings from these studies are summarized in Table 1.

**Table 1:** Summary of previous studies (*N* = 19) on the relation between racial discrimination and brain activity

Study	Region	Research method	Sample	Racial discrimination measure	MRI/fMRI condition	MRI/fMRI measure	ROIs	Covariates	Key findings
Beatty Moody et al. (2019)	USA	Cross-sectional study	N = 71 African Americans (*M*_age_ = 50.58, *SD* = 9.92); 60.6% female)	Lifetime discrimination burden (one item from Experiences of Discrimination Questionnaire) and racial discrimination (Krieger et al., 2005)	Structural MRI	Brain volume	Total white matter lesion volume (WMLV)	Age, sex, and socioeconomic status in the interaction analysis	Significant interactions between age, lifetime discrimination burden, and racial discrimination in relation to white matter lesion volume (WMLV) were found. Higher levels of both lifetime and racial discrimination were associated with greater WMLV among older African Americans. Lower levels of racial discrimination showed increased WMLV among younger African Americans.
Chen et al. (2025)	USA	Longitudinal (baseline and 2-year follow-up)	N = 3,321 (43% Hispanic, 30% Black, 23% Asian, and 4% other; 48% female; *M*_age_ = 9.47, *SD* =.51)	Seven child-reported items on perceptions of racial discrimination	Resting-state fMRI	Functional connectivity	13 large-scale networks based on Gordon parcellation, amygdala, hippocampus, and nucleus accumbens	Sex in the mediation moderation analysis	Racial discrimination predicted 2-year decreases in nucleus accumbens-retrosplenial/sensorimotor and amygdala-sensorimotor connectivity and increased hippocampal–auditory connectivity. Racial discrimination was linked to weaker right nucleus accumbens-retrosplenial connectivity and greater externalizing symptoms. Sex moderated effects: weaker connectivity predicted higher internalizing symptoms among girls. Greater racial discrimination was associated with weaker connectivity, and increased internalizing symptoms among girls.
Clark et al. (2018)	USA	Cross-sectional study	N = 74 (71.6% African-America/Black, 9.5% Caucasian/White, 9.5% Bi/Multiracial, 1.4% Asian American, 8.1% Other; *M*_age_ = 47.46, *SD* = 10.95; 56.8% male)	The Everyday Discrimination Scale (EDS)	Resting-state fMRI	Functional connectivity	Amygdala	Stress, depression, anxiety, PTSD, HIV status, age, sex, race, ethnicity, sexual orientation, and urine toxicology	Increased racial discrimination was related to heightened spontaneous activity in the amygdala. Increased racial discrimination was associated with functional connectivity between amygdala and brain regions (e.g., thalamus, anterior insula, putamen, caudate, anterior cingulate, medial frontal gyrus). These effects were significant after including control variables (i.e., age, race, stress, PTSD, depression, etc.).
Elbasheir et al. (2024a)	USA	Correlational study	72 Black women (*M*_age_ = 38.17, *SD* = 10.98)	Experiences of Discrimination (EOD) questionnaire	Task-based fMRI; Affective Stroop task during fMRI	Functional connectivity	The bilateral insula (anterior and posterior regions) and amygdala	Age and scanner	During attention to threat-related affective Stroop trials, individuals with higher levels of racial discrimination showed reduced connectivity between the insula and multiple clusters in the medial prefrontal cortex (mPFC). The strength of insula-mPFC connectivity was significantly and negatively correlated with derealization symptoms, but not with PTSD symptoms.
Elbasheir et al. (2024b)	USA	Cross-sectional study	40 Black American women (*M*_age_ = 39.9, *SD* = 10.0)	The Experiences of Discrimination (EOD) questionnaire	Task-based fMRI; Affective Number Stroop task (trauma-relevant and natural-relevant trials)	BOLD	The left and right middle occipital gyrus, and ventromedial prefrontal cortex	Latency between blood draw and age	Racial discrimination was not associated with the ventromedial prefrontal cortex (vmPFC), left and right middle occipital cortex activation during attention to threat. Participants with high C-reactive protein (CRP) levels exhibited a positive relation between vmPFC and racial discrimination.
Elbasheir et al. (2024c)	USA	Cohort study	90 Black women (*M*_age_ = 38.5, *SD* = 11.3)	The Experiences of Discrimination (EOD) questionnaire	Resting-state fMRI	Functional connectivity	Brainstem and midbrain (bilateral locus coeruleus, periaqueductal gray, and bilateral superior colliculus)	The traumatic events inventory frequency, PTSD scores, and age	Racial discrimination was associated with increased brainstem and midbrain resting-state functional connectivity (RSFC) between the left locus coeruleus (LC) and the bilateral precuneus. No relation between racial discrimination and right LC RSFC was observed. The left LC RSFC mediated the relation between racial discrimination and DNA methylation age acceleration.
Fani et al. (2021)	USA	Cross-sectional study	55 trauma-exposed US Black women (*M*_age_ = 37.7, *SD* = 10.7)	Experiences of Discrimination (EOD) questionnaire	Task-based fMRI; Affective Stroop task (trauma-related and natural-related trials), fMRI	BOLD	Whole brain	The traumatic events inventory frequency and PTSD scores	Experiences of racial discrimination were associated with increased BOLD responses in middle occipital cortex and ventromedial prefrontal cortex during trauma-related distractor trials. The effects remained comparable after adjusting for trauma and PTSD symptoms.
Fani et al. (2022b)	USA	Correlational study	116 Black American women (*M*_age_ = 39.0, *SD* = 11.8)	Experiences of Discrimination (EOD) questionnaire	Resting-state Diffusion tensor imaging	Diffusion tensor imaging; fractional anisotropy from white matter.	Inferior longitudinal fasciculus, superior longitudinal fasciculus, uncinate fasciculus, Cingulum (anterior and posterior), Corpus Callosum, and Fornix	PTSD score, the traumatic events inventory score, monthly income, and Scanner location in the initial analysis. Age in the follow-up analysis.	Experiences of racial discrimination were associated with decreased fractional anisotropy in white matter tracts of the corpus callosum, cingulum, and superior longitudinal fasciculus. The effects were consistent after adjusting for covariates such as trauma, posttraumatic stress disorder, and demographic factors.
Fani et al. (2022a)	USA	Cross-sectional study	81 Black American women (*M*_age_= 39.3, *SD* = 11.8)	The Experiences of Discrimination (EOD) questionnaire	Resting-state structural MRI	Cortical thickness, surface area, and gray matter volume	Cingulate cortex regions [left and right rostral anterior cingulate cortex (rACC), caudal anterior cingulate cortex (cACC), and posterior cingulate cortex (PCC)], Amygdala and hippocampal volume	PTSD score, intracranial volume, childhood trauma questionnaire score, traumatic events inventory adult trauma score, monthly household income in the main analysis. Age for the re-analysis.	Racial discrimination was associated with lower cingulate cortex thickness. No statistically significant association of racial discrimination with cingulate cortex surface or volume was found. No relation between racial discrimination and gray matter subcortical (amygdala and hippocampus) was observed.
Han et al. (2021)	USA	Cross-sectional study	124 Black adults (*M*_age_ = 74.9, *SD* = 6.3; 85.6% women)	Self-Reported Experiences of Discrimination	Resting-state fMRI	Functional connectivity	The left and right amygdala and left and right insula	Age, education, sex, and global cognitive function	Racial discrimination was associated with greater the left insula and the bilateral intracalcarine cortex connectivity, lower the left insula and the left dorsolateral prefrontal cortex connectivity, and lower the right insula and the left supplementary motor area connectivity. No relation was found between racial discrimination and amygdala.
Kral et al. (2024)	USA	Longitudinal study - cross-sectional data was reported	Mother-infant dyads (N = 25; pregnant *M*_age_ = 28.5, *SD* = 6.2). Infants *M*_age_ at the time of the MRI = 13.5 days, *SD* = 2.45 days; 52% female; 21 Black, 1 Asian, and 3 other races).	Experiences of Racism Scale (REQ).	Resting-state fMRI	Functional connectivity	Right and left amygdala and hippocampus	Infant sex and gestational age at the 2-week MRI session were included in all analyses. Additional covariates included maternal perceived stress, maternal income-to-needs ratio, and the brief symptom inventory score.	Greater maternal experiences of racism were linked to stronger resting-state functional connectivity in the right amygdala, specifically with clusters in the primary visual cortex and thalamus. Increased maternal experiences of racism were positively correlated with stronger resting-state functional connectivity in the left hippocampus, particularly with clusters in the primary visual cortex (calcarine), middle and superior parietal cortex, and superior temporal cortex. Additionally, there was a positive association with stronger resting-state functional connectivity in the right hippocampus, linking to clusters in the superior temporal cortex, precuneus, and left parahippocampus.
Masten et al. (2011)	USA	Cross-sectional study	18 Black individuals (*M*_age_ = 21.4; 50% women)	Self-reported discriminatory attributions	Task-based fMRI; Cyberball paradigm	BOLD	Whole brain	No covariates	Racial discrimination (exclusion because of their race) was associated with lower activity in the areas related to social threats (i.e., dorsal anterior cingulate cortex and motor cortex) and greater activity in the areas related to regulation of threat responses (i.e., rostral anterior cingulate cortex, lateral temporal cortex, and brainstem). Reverse outcomes were observed for the relation between distress and neural activities.
Meyer et al. (2019)	USA	Cross-sectional study	710 participants (*M*_age_ = 50.3, *SD* = 3.5; 53% women; 286 Black participants)	The Experience of Discrimination Index	Resting-state structural MRI	Brain tissue volumes (white matter, gray matter, and cerebrospinal fluid).	Abnormal tissue volumes for gray and white matter for all regions	Sex, age, alcohol, smoking status, education, body mass index, hypertension, diabetes, study site, and intracranial volume	Lower levels of brain matter volume among Black participants who experienced both depression and racial discrimination compared to those with no experience. Lower levels of brain matter volume among Black participants who experienced both depression and racial discrimination compared to those with only experiences of racial discrimination. Lower levels of white matter volume among Black participants who experienced both depression and racial discrimination compared to those with no experience, and only experiences of racial discrimination, and or only experience of depression. No differences were found among White participants.
Mullins et al. (2024)	USA	Cross-sectional study	N = 30 Latina girls (*M*_Age_ = 9.76, *SD* = 1.11, range = 8-12 years)	The Perceptions of Racism in Children and Youth (PRaCY; Pachter et al. 2010)	Resting-state structural MRI	Brain volume	The left and right amygdala	Total intracranial volume and annual household income	Participants with higher levels of ethnic-racial discrimination had reduced left amygdala volume compared to those with lower levels of ethnic-racial discrimination controlling for total intracranial volume and annual household income. No relation was found for the right amygdala.
Oshri et al. (2024)	USA	Cross-sectional study	1596 Black youths (50.3% female; *M*_age_ = 10.92, *SD* =.63)	The 7-item discrimination questionnaire (interpersonal racial discrimination and feelings of marginalization)	Task-based fMRI: emotional N-Back task (emotional faces)	Not mentioned	Amygdala	Age, biological sex, and income	Interpersonal racial discrimination was not directly associated with amygdala responses to negative emotional faces. No moderation role of amygdala was observed for the relation between interpersonal racial discrimination and internalizing/externalizing symptoms.
Okeke et al. (2023)	USA	Correlational study	79 Black American women (*M*_age_ = 39.7, *SD* = 11.3 years)	Experiences of Discrimination (EOD) questionnaire	Resting-state Diffusion tensor imaging	Diffusion tensor imaging; fractional anisotropy from white matter pathway.	Corpus Callosum, Superior Longitudinal Fasciculus, and Anterior Cingulum Bundle	The traumatic events inventory score, scanner site, and income	Racial discrimination was negatively associated with left anterior cingulum and genu corpus callosum. Racial discrimination was associated with increased health problems. No significant relation between racial discrimination and superior longitudinal Fasciculus was observed.
Webb et al. (2022)	USA	Cross-sectional study	102 Black adults (*M*_age_ = 33, *SD* = 10; 58% women)	Perceived Ethnic Discrimination Questionnaire (PEDQ)	Resting-state fMRI	Functional connectivity	The anterior insula and amygdala	Scores on the life events checklist for DSM-5, PTSD, and income	Racial discrimination was associated with greater Amygdala-thalamus and Insula-thalamus connectivity after adjusting for PTSD, income, and life events.
Wright et al. (2020)	USA	Cross-sectional study	24 African Americans (*M*_age_ = 51, *SD *= 14.6; 54% women)	The Everyday Discrimination Scale	Task-based fMRI; videos were presented (emotional, analytic, and practical self-care instructions combined both analytic and emotional)	BOLD	Medial parietal cortex and neighboring posterior cingulate, the dorsal medial prefrontal cortex, temporoparietal junction, the lateral parietal, lateral prefrontal cortices, and the ventral medial prefrontal cortex	No covariates	Racial discrimination was associated with the empathy network (i.e., the medial parietal cortex and neighboring posterior cingulate, the dorsal medial prefrontal cortex, and temporoparietal junction) and the ventral medial prefrontal cortex (vmPFC). No significant relation between racial discrimination and the analytic network was found.
Zahodne et al. (2023)	USA	Longitudinal study	221 non-Hispanic Black participants (*M*_age_ = 73.5, *SD* = 6.0; 66.1% female)	Everyday Discrimination and Major Experiences of Lifetime Discrimination	Resting-state structural MRI	Brain volume	Hippocampal and white matter hyperintensity volumes	Sex/gender, age, education, income, total intracranial volume at the initial scan (this covariate was only included in the outcome), and time between scans (this covariate was only included in the latent changes of the outcome)	Lifetime racial discrimination was associated with decreased initial hippocampal volume. Everyday racial discrimination was associated with faster accumulation of white matter hyperintensity over time. Neither everyday nor lifetime racial discrimination was statistically associated with subsequent changes in hippocampal volume.

## Discussion

### Functional and structural MRI studies

Regarding resting-state fMRI/MRI, 13 studies were found (Beatty Moody et al., 2019; Chen et al., 2025; Clark et al., 2018; Elbasheir et al., 2024c; Fani et al., 2022a, b; Han et al., 2021; Kral et al., 2024; Meyer et al., 2019; Mullins et al., 2024; Okeke et al., 2023; Webb et al., 2022; Zahodne et al., 2023). These studies have considered different brain regions to investigate functional connectivity using fMRI (Chen et al., 2025; Clark et al., 2018; Elbasheir et al., 2024c; Han et al., 2021; Kral et al., 2024; Webb et al., 2022), fractional anisotropy from white matter pathway using diffusion tensor imaging (Fani et al., 2022b; Okeke et al., 2023), and brain volume using structural MRI (Beatty Moody et al., 2019; Fani et al., 2022a; Meyer et al., 2019; Mullins et al., 2024; Zahodne et al., 2023). In this part, we discuss findings based on the neuroimaging measurement methods reported by previous studies.

### Resting-State functional connectivity

A growing body of neuroimaging research has explored how experiences of racial discrimination may alter patterns of brain functional connectivity within brain networks. The functional connectivity refers to statistical connection between brain functions/activities/signals of two distinct brain regions (Mohanty et al., 2020). Collectively, these studies demonstrate that racial discrimination is associated with significant alterations in intrinsic resting-state functional connectivity (rsFC). Findings indicate that experiences of racial discrimination are not only an important factor in early brain development (Kral et al., 2024), but also it is crucial in biological aging (Elbasheir et al., 2024c). In this line, Kral et al. (2024) conducted a study on 25 mother-infant dyads (21 Black, 1 Asian, and 3 other races) in the US to examine the intergenerational impact of maternal experiences of racism during pregnancy on infant brain development. The infants had resting-state fMRI brain scans at approximately 2 weeks of age. Findings revealed that greater maternal experiences of racism were associated with stronger rsFC in the right amygdala, particularly with regions, including the primary visual cortex and thalamus. Similarly, increased maternal racism exposure was linked to heightened connectivity in the left hippocampus, especially with the primary visual cortex (calcarine), middle and superior parietal cortex, and superior temporal cortex. Stronger connectivity in the right hippocampus was also observed, with significant links to the superior temporal cortex, precuneus, and left parahippocampus. These results suggest that maternal experience of racism could be associated with the brain network related to emotional memory and vigilance among racialized groups. However, given the small sample size and cross-sectional data, it is unclear whether these patterns reflect temporary states in neonatal brain function or longer-term neural function. Interpretation should therefore remain cautious, and replication in larger, longitudinal samples is needed to determine the durability and functional significance of these associations.

Concerning ageing, Elbasheir et al. (2024c) showed that racial discrimination was associated with increased rsFC between the left locus coeruleus and bilateral precuneus, which is related to rumination. This rsFC mediated the relationship between racial discrimination and DNA methylation age acceleration among Black women. They measured DNA methylation age acceleration using peripheral blood, which is used to predict advanced cellular aging. These findings indicate a role of racial discrimination in the brain networks related to cellular aging and potentially neurodegenerative disease.

It is also suggested that racial discrimination is linked to increased functional connectivity between amygdala and insula, and several brain regions that are implicated in emotion regulatory system (Clark et al., 2018; Han et al., 2021), sensitivity to threats and adaptation process (Webb et al., 2022), and trust perception (Han et al., 2021). Clark et al. (2018) conducted a cross-sectional study to investigate the neural correlates of discrimination among racial groups (71.6% African-America/Black, 9.5% Caucasian/White, 9.5% Bi/Multiracial, 1.4% Asian American, 8.1% Other). Results indicated that higher levels of discrimination were significantly associated with increased spontaneous activity in the amygdala, a region central to emotional and threat processing. Furthermore, greater discrimination was linked to heightened functional connectivity between the amygdala and several brain regions implicated in emotion regulation and salience detection, including the thalamus, anterior insula, putamen, caudate, anterior cingulate cortex, and medial frontal gyrus. These associations remained robust after controlling for age, race, perceived stress, PTSD symptoms, and depressive symptoms, highlighting the persistent impact of discrimination on brain function at rest. However, they did not disaggregate the results by racial groups and discrimination was assessed broadly rather than capturing race-specific experiences.

Moreover, Han et al. (2021) investigated the relation between experiences of racial discrimination and insula functional connectivity among older Black adults. They found that racial discrimination was associated with increased connectivity between the left insula and bilateral intracalcarine cortex, and decreased connectivity between the left insula and left dorsolateral prefrontal cortex (dlPFC), as well as between the right insula and left supplementary motor area. The connectivity between the left insula and the bilateral intracalcarine cortex may reflect a proactive, adaptive mechanism for social surveillance, enabling rapid, intuitive (“gut”) evaluation of potentially discriminatory social environments. This enhanced integration may support vigilance and quick threat appraisal. Decreased connectivity between the left insula and dlPFC suggests diminished top-down regulation of interoceptive and emotional responses. This could reflect a release of regulatory control, leaving the insula more autonomous in evaluating trust and social cues, potentially leading to hypervigilance or emotional overload. Lastly, reduced connectivity between the right insula and left supplementary motor area (SMA) may point to possible disruptions in linking emotional or social perception with motor planning. Moreover, older Black adults with increased experiences of racial discrimination may have developed adaptive strategies to suppress “gut” emotional responses from physical action that might explain the observed weaker connectivity between the insula and regions involved in motor planning (Han et al., 2021).

Similarly, Webb et al. (2022) tested the association between racial discrimination and brain activities among Black adults who experienced a traumatic injury. They showed that racial discrimination was associated with greater connectivity between the bilateral amygdala-thalamus and bilateral anterior insula-precuneus after adjusting for PTSD, income, and life events. These findings suggest that the increased amygdala-thalamus connectivity is linked to sensitivity to threats and might be associated with adaptive coping to threats related to racial discrimination. That is, sustained engagement in coping strategies to manage racism may result in neural vulnerabilities or heightened biological sensitivity, especially when compounded by acute trauma, leading to mental health issues (Forsyth & Carter, 2012; Webb et al., 2022). Moreover, insula-precuneus connectivity is linked to general arousal and enhanced readiness to process biologically relevant information. Webb et al. (2022) mentioned that the insula-precuneus connectivity plays an important role in mental imagery, self-awareness, and recollection of past experiences.

Extending these findings to adolescence, the most recent study, conducted by Chen et al. (2025), on 3,321 racial/ethnic minority youth focused on neural mechanisms of racial discrimination and its mental health outcomes. They showed that racial discrimination was associated with 2-year changes in decreased connectivity between the nucleus accumbens and retrosplenial-temporal and sensorimotor networks, decreased amygdala-sensorimotor connectivity, and increased hippocampal-auditory network connectivity. These findings highlight accelerated maturation in the observed brain connections. They also developed a moderated mediation model, where racial discrimination was the predictor, nucleus accumbens (right)-retrosplenial-temporal connectivity was the mediator, sex was the moderator, and internalizing and externalizing symptoms were the outcome variables. Their results showed no mediation role of the brain connectivity; however, racial discrimination was negatively associated with nucleus accumbens (right)-retrosplenial-temporal connectivity and positively associated with externalizing symptoms. Sex moderated the relation between nucleus accumbens(right)-retrosplenial temporal connectivity and internalizing and externalizing symptoms, where weaker connectivity was associated with increased internalizing symptoms among girls. They also found that greater racial discrimination was associated with weaker connectivity, and increased internalizing symptoms among girls. These findings highlight racial discrimination uniquely interrupts developing brain circuits in adolescent girls, which may indicate a sex-specific neural pathway that increases vulnerability to internalizing symptoms.

In sum, converging evidence from neuroimaging studies highlights that racial discrimination significantly alters rsFC across brain networks involved in emotional processing, vigilance, memory, and self-awareness. These patterns are consistent with the hypothesis proposed by Muscatell et al. (2022), suggesting that racial discrimination may be linked to networks underlying affective processing and memory, and cognitive control. These changes are evident across the lifespan, from early infancy to older adulthood, and are associated with both adaptive and maladaptive neural responses. Increased connectivity in salience and visual networks may reflect heightened readiness to detect threats, while weakened top-down regulatory pathways suggest potential neural vulnerabilities. These findings indicate the profound impact of racism on brain development, functioning, and aging, which may result in mental and physical health issues among Black individuals. The presence of these alterations from early infancy through older adulthood aligns with the developmental and longitudinal perspective central to the CoRT model, supporting the conceptualization of racial discrimination as a chronic and cumulative traumatic stressor with potential intergenerational effects.

#### Fractional Anisotropy

One key focus in neuroimaging is the alteration of white matter microstructure, often measured by fractional anisotropy (FA) (Ikenouchi et al., 2020; Salat et al., 2005; Takao et al., 2013). Studies exploring the relationship between racial discrimination and white matter pathways have highlighted changes in FA as a key marker of structural brain alterations. Fani et al. (2022b) conducted a study on the relation between racial discrimination and white matter microstructure among 116 Black American women. They showed that racial discrimination was associated with decreased fractional anisotropy in the corpus callosum, cingulum bundle, and superior longitudinal fasciculus. These effects remained significant after controlling for trauma, PTSD, and demographic factors, suggesting that the independency of racial discrimination related to the brain structure. The corpus callosum serves as the brain’s primary pathway for interhemispheric communication which is sensitive to the effects of early-life adversity (Jackowski et al., 2008). The superior longitudinal fasciculus is a key frontoparietal white matter tract involved in various cognitive and emotional processes (Nakajima et al., 2020). As well, the cingulum bundle is a major fiber tract that connects the parahippocampal regions with the parietal and prefrontal cortices, which plays a critical role in executive function (e.g., inhibition) and emotional processing (Fani et al., 2015; Heilbronner & Haber, 2014). These pathways are associated with PTSD (Daniels et al., 2013; Siehl et al., 2018; Suo et al., 2023; Weis et al., 2018). Fani et al. (2022b) stated that prolonged exposure to racial discrimination may weaken the cingulum bundle, resulting in problems in inhibitory control system against threats. Consistent with the Fani et al. (2022b) study, Okeke et al. (2023) showed that racial discrimination was negatively associated with the left anterior cingulum and genu corpus callosum and positively associated with increased health problems. However, their results regarding the superior longitudinal fasciculus were not consistent with Fani et al. (2022b) study, where Okeke et al. (2023) showed no significant relation between the superior longitudinal fasciculus and racial discrimination. They also showed a significant indirect effect of racial discrimination on health problems through left anterior cingulum bundle fractional anisotropy and corpus callosum genu fractional anisotropy. These findings indicate that integrity of stress-sensitive prefrontal white matter tracts could contribute to health problems.

These results suggest that structural changes in important white matter tracts that are involved in emotion regulation, cognitive control, and interhemispheric communication may reflect the effects of racial discrimination. The consistent associations between decreased FA in the cingulum bundle and corpus callosum across studies suggest a potential neurobiological imprint of chronic racial stress/race-based stress. Within the CoRT model, such patterns are consistent with the cumulative neurobiological embedding of prolonged, multilevel stress exposure. Moreover, these results may open more research questions underlying a form of stress grounded in racial discrimination as proposed by Moshirian Farahi & Cénat (2026). Furthermore, the connection between these brain alterations and physical health consequences emphasizes the wider effects of stress grounded in racial discrimination on the brain and body systems. All of this evidence points to racial discrimination as a quantifiable neurobiological stressor in addition to a social and psychological burden.

#### Brain volume

Studies investigating the impact of racial discrimination on brain structure indicate that discrimination may affect both cortical and subcortical areas, although the results are complex. Direct correlations between discrimination and volumetric measures suggest that certain brain regions may exhibit heightened sensitivity to racial stress. For example, Fani et al. (2022a) investigated the relation between racial discrimination and cingulate cortex thickness among 81 trauma-exposed black women. They showed that racial discrimination was negatively associated with thickness of the left rostral anterior cingulate cortex (rACC), left caudal anterior cingulate cortex (cACC), and left posterior cingulate cortex (PCC) after controlling for trauma exposure, PTSD, total intracranial volume, and income. However, no relation between racial discrimination and other area of interests (i.e., cingulate, amygdala, and hippocampal volumes) was found after controlling for covariates. These findings indicate the important role of cortical thickness of anterior and posterior cingulate cortex in brain vulnerability related to racial discrimination.

Despite Fani et al. (2022a), Zahodne et al. (2023), a longitudinal study on 221 non-Hispanic older Black adults, found that lifetime racial discrimination was associated with decreased initial hippocampal volume, while everyday racial discrimination was not associated with neither initial nor later changes. Everyday racial discrimination was linked to faster accumulation of white matter hyperintensity (WMH) over time. However, no significant associations were found with subsequent changes in hippocampal volume. Lifetime racial discrimination was not associated with initial WMH volume or change in WMH volume. The association between racial discrimination and change in WMH volume can contribute to health outcomes such as systemic inflammation and cardiovascular issues (Beatty Moody et al., 2019; Zahodne et al., 2023). These findings reflect the importance of racial discrimination in cognitive aging among Black individuals.

Evidence for volumetric effects of discrimination alone, however, is not entirely consistent. For instance, early research conducted by Meyer et al. (2019) showed that lower levels of brain matter volume among Black participants who experienced both depression and racial discrimination compared to those with no experience and those with only experiences of racial discrimination. Moreover, they found lower levels of white matter volume among Black participants who experienced both depression and racial discrimination compared to those with no experience, and only experiences of racial discrimination, and or only experience of depression. These findings indicate that although racial discrimination, only, was not associated with abnormal white matter tissue, it was evident that combined depression and racial discrimination was associated with abnormal white matter volume among Black individuals. Beatty Moody et al. (2019) did not find a main effect of lifetime racial discrimination burden or racial discrimination on white matter lesion volume (WMLV). However, the interaction between both measure of racial discrimination and age on WMLV was significant, where increased lifetime discrimination burden and racial discrimination were associated with increased WMLV among older African Americans, while this effect was negative among younger African Americans. This relationship could explain risks for stroke, dementia, and other brain-related health issues.

While there were 4 studies among Black individuals, there was only one study that investigated the relationship between ethnic-racial discrimination exposure and amygdala volume among Latina girls aged 8-12 (Mullins et al., 2024). Mullins et al. (2024) showed that participants with higher levels of ethnic-racial discrimination had reduced left amygdala volume compared to those with lower levels of ethnic-racial discrimination controlling for total intracranial volume and annual household income. No relation was found for the right amygdala. Their mediational analysis showed that left amygdala mediated the relation between ethnic-racial discrimination and anxiety.

In sum, these findings suggest that racial discrimination can shape brain structure, particularly in the cingulate cortex, hippocampus, and amygdala. However, these effects are not uniformly observed, with some studies indicating that racial discrimination alone may not be sufficient to produce volumetric changes. Instead, as proposed by the CoRT model, these effects may be context-dependent, emerging in combination with other stressors (e.g., depression, trauma) or cumulative over the lifespan, underscoring the complex pathways linking social adversity to neurobiological outcomes.

### Task-based MRI studies

Task-based functional MRI paradigms shed light on how racial discrimination experiences affect context-dependent neural reactivity during threat processing, social evaluation, emotion regulation, and cognitive control. A total of six studies has used tasks during the MRI scanning (Elbasheir et al., 2024a, b; Fani et al., 2021; Masten et al., 2011; Oshri et al., 2024; Wright et al., 2020). The very first study on the racial discrimination and MRI scanning during a task on 18 Black individuals was conducted by Masten et al. (2011). They used a Cyberball task, which has been used to induce experience of social exclusion (inclusion vs. exclusion). They found that racial discrimination (exclusion due to race) was associated with lower activity in areas related to threats and distress (dorsal anterior cingulate cortex and motor cortex) and greater activity in areas related to emotion regulation (rostral anterior cingulate cortex, lateral temporal cortex, and brainstem). In contrast, experience of distress was associated with increased activity in social-pain related areas and lower activity in areas related to emotion regulation. Masten et al. (2011) concluded that individuals regularly facing negative social treatment manage to cope with immediate experiences related to discrimination, however, long-term effects require further research.

Subsequent studies using affective paradigms have further tested racial discrimination-related modulation of threat and regulatory circuits. Fani et al. (2021) conducted a cross-sectional study on 55 trauma-exposed Black American women. Functional MRI was recorded during an affective Stroop task (i.e., trauma-related, positive, and natural distractors). They found that racial discrimination was associated with increased blood oxygenation level-dependent (BOLD) responses in the middle occipital cortex and ventromedial prefrontal cortex during trauma-related distractor trials. These effects remained significant after adjusting for trauma and PTSD symptoms. These results indicate that racial discrimination is linked to the areas related to fear inhibition, emotion regulation, and visual attention. They mentioned that exposure to racial discrimination could lead to elevated attentional responses to trauma-related distractors and regulatory responses. Likewise, Elbasheir et al. (2024b) used an affective number Stroop task among 40 Black American women. However, they found no association between racial discrimination and activation in the ventromedial prefrontal cortex or the left and right middle occipital cortex during attention to threat. They also included C-reactive protein levels (measure of inflammation) to test its moderation effect on the relation between vmPFC activation and racial discrimination. They showed that participants with high C-reactive protein levels showed a positive relation between vmPFC activation and racial discrimination. These findings suggest that the moderation role of C-reactive protein on vmPFC activation may play an important role in emotion regulation. That is, experiences of racial discrimination could contribute to vmPFC inflammation, resulting in increased response to threat and changes in emotion regulation circuitry. Elbasheir et al. (2024a) published another paper investigating the neural mechanisms linking racial discrimination to emotion regulation and dissociative symptoms in a sample of 72 Black women. They conducted task-based functional magnetic resonance imaging (fMRI) while performing an affective Stroop task designed to probe attentional responses to threat-related stimuli. This study focused on functional connectivity involving the bilateral insula (including both anterior and posterior regions) and the amygdala. Results showed that higher reported experiences of racial discrimination were associated with reduced connectivity between the insula and clusters in the medial prefrontal cortex (mPFC) during attention to threat-related trials. Furthermore, the strength of insula-mPFC connectivity was significantly and negatively correlated with derealization symptoms, suggesting a potential neural pathway through which discrimination may contribute to dissociative experiences. Notably, this relationship was not observed for PTSD symptoms, indicating a more specific link to dissociation rather than general trauma-related distress.

Evidence based on tasks also applies to more general processing. Wright et al. (2020) tested the relation between neural processing of health information and perceived discrimination among 24 African Americans. They used video presentations of emotional, analytic, and practical self-care instructions while the brain activities were being recorded. They found that racial discrimination and global stress was associated with activation in the empathy network (medial parietal cortex, posterior cingulate, dorsal medial prefrontal cortex, and temporoparietal junction) and ventral medial prefrontal cortex, but not with the analytic network. Their findings indicate that these regions are important in stress regulation and motivation to change behaviors.

Unlike adult samples where heightened regulatory recruitment is often observed, large-scale adolescent data suggest more nuanced or sex-dependent amygdala patterns. In this line, the most recent study, performed by Oshri et al. (2024), examined how interpersonal racial discrimination and feeling of marginalization is associated with mental health outcomes through amygdala response to negative facial valence using an emotional N-Back task among 1,596 Black youths (mean_age_ = 10.92) in the US. Neither interpersonal racial discrimination nor feeling of marginalization was associated with amygdala responses to negative facial emotion. No moderation role of amygdala was observed for the relation between interpersonal racial discrimination and internalizing/externalizing symptoms. However, for feeling of marginalization, deactivation right amygdala amplified internalizing symptoms but buffered against externalizing symptoms, whereas high left amygdala activation was protective, decreasing externalizing symptoms.

In sum, task-based fMRI studies indicate that racial discrimination is associated with brain areas related to emotion regulation, threat detection, and attentional control. These findings are consistent with the CoRT model, where racial discrimination may be associated with several emotional and cognitive outcomes. Results show disturbed connectivity between prefrontal networks and salience regions (insula, amygdala) and altered prefrontal recruitment. This finding is also consistent with the hypothesis that racial discrimination is linked to affective processing involved in the salience network (Muscatell et al., 2022). Although marginalization had complex effects, there was no clear correlation between racial discrimination and amygdala reactivity in youths. Overall, these studies show that racial discrimination may have both short-term adaptive and long-term psychological effects; however, more research is required to explain the biological moderators and causal mechanisms.

## Limitations

Recent studies on racial discrimination and its impact on neurobiological outcomes have highlighted various limitations that need to be addressed in future research (Table 2). Several studies relied on cross-sectional designs, limiting the ability to draw causal inferences. For instance, some studies (Elbasheir et al., 2024b, c) acknowledged that cross-sectional data prevent conclusions about the directionality of relationships between racial discrimination and neural outcomes. Moreover, issues such as lack of longitudinal follow-up were noted by researchers (Beatty Moody et al., 2019; Zahodne et al., 2023). The absence of longitudinal studies further restricts the ability to examine cumulative effects or potential recovery processes over time. Cumulative exposure to racism may result in racial trauma (Cénat, 2023); however, the neurobiological mechanisms underlying this cumulative process remain largely unexplored.

**Table 2:** Limitations reported by the previous studies

Study	Limitations
Beatty Moody et al. (2019)	Lack of longitudinal data, potential recall bias due to using self-reports, racial discrimination data was collected 5 years earlier than MRI scan, small sample size, limited cognitive data to test correlations, and use of one single item for racial discrimination burden.
Chen et al. (2025)	No functional inferences using behavioural tasks, no assessment of institutional/systemic racism, no examination of perpetrator roles (e.g., teachers), cross-sectional (early adolescence only), limited subgroup (racial/ethnic, sex) analyses, retrospective reporting of discrimination, lack of timing precision (discrimination vs. brain changes). limited focus on protective/modifiable factors, exclusion of some sub-groups (e.g., non-Hispanic Black participants, unmarried) given the missing observations.
Clark et al. (2018)	Cross-sectional design, using self-reports to measure racial discrimination, unexplored factors such as resilience, coping, and distress tolerance in the effects, and comparable to previous studies a 5-min resting-state was recorded, however, longer recording could increase reliability.
Elbasheir et al. (2024a)	Cross-sectional design, sample limited to women, racial discrimination was measured as a general measure, limited brain regions were tested, and small sample size, leading to limited statistical power.
Elbasheir et al. (2024b)	No causal inference (cross-sectional study), limited brain region analysis (i.e., brainstem and midbrain areas), lack of measure of coping responses, potential recall bias regarding racial discrimination, measuring DNA methylation with peripheral blood and small sample size for DNA methylation, and sample was limited to women.
Elbasheir et al. (2024c)	Cross-sectional, no gender differences due to limited sample to women, insula subregion analysis was not conducted (e.g., posterior, anterior, etc.), and lack of interoception and dissociation measurement during scanning.
Fani et al. (2021)	Sample limited to Black women, no investigation on the effect of racial discrimination on stress regulation and immune system, general investigation on racial discrimination (i.e., different racist acts should be investigated), and absence of experimental manipulations (e.g., the effect of real-time experiences of discrimination)
Fani et al. (2022b)	Single assessment of racial discrimination and not assessing structural racism, unimodal approach to brain structure by focusing on white matter metrics, lack of intersectional analysis, sample was limited to women, did not investigate the relation between white matter integrity and health outcomes.
Fani et al. (2022a)	Cross-sectional design, difficulty distinguishing stressor effects that concurrent with racial discrimination for testing their effect on the brain, no assessment of intersectional discrimination (e.g., gendered racism), and limited gender diversity to women.
Han et al. (2021)	Cross-sectional design, using self-reported discrimination, lacks information on the emotional or psychological intensity of reported discrimination, low reported discrimination which may reflect scale characteristics or cohort effects, limited brain areas, and unclear whether the reported discrimination was race-based.
Kral et al. (2024)	Small sample size, had more Black people in the sample, using self-reported scale may lead to recall bias, and potential moderation effects and unexplored mechanisms (e.g., glucocorticoid exposure, salience network dynamics, etc.)
Masten et al. (2011)	All researchers/interviewers who asked about exclusion experience were White, not interview recorded video before scanning (absence of baseline affect data), and uncertainty in interpreting neuroimaging results (i.e., due to the multifunctionality of brain regions, it is difficult to draw definitive conclusions without direct testing).
Meyer et al. (2019)	Cross-sectional design, participants may have underreported discrimination experiences, limited generalizability to full cohort due to exclusion criteria.
Mullins et al. (2024)	Small sample size, cross-sectional study, limited to structural MRI which could not present functional data, observed high levels of anxiety among this community-based sample may limit generalizability.
Oshri et al. (2024)	Preliminary study requires replication, need to control for false discovery rate, no modeling of location-based variability in racism, no analysis of intersectionality and to consider complexity of racism, focused on amygdala.
Okeke et al. (2023)	Cross-sectional design, limited to women, lack of intersectional analysis, health conditions were combined to preserve statistical power, limiting disorder-specific insights, no data on health outcomes (e.g., inflammation) that might explain links between racism and white matter changes.
Webb et al. (2022)	Cross-sectional design, homogeneous sample (i.e., people experienced a motor vehicle crash), confounding trauma sources (past racial trauma, current, or other lifetime PTSD), and lack of measuring protective factors such as resilience or coping.
Wright et al. (2020)	A secondary analysis, small sample size and moderate effect size, and no other ethnic group or gender analysis.
Zahodne et al. (2023)	Timing of discrimination assessments which occurred after the first MRI, limited follow-up MRI data due to COVID-19 disruptions, participants were all non-Hispanic Black older adults from northern Manhattan, limiting generalizability to other regions or rural settings.

Furthermore, many studies had limited sample diversity, often focusing on Black women or other specific demographic groups (Elbasheir et al., 2024a, b, c; Fani et al., 2021, 2022a; b; Okeke et al., 2023), which may restrict the generalizability of the findings to other racial, ethnic, or gender groups. However, we acknowledge that such single-race or demographically focused studies remain important for characterizing group-specific associations and partially addressing the underrepresentation of racialized populations in the literature. In addition, small sample sizes were a common issue (Beatty Moody et al., 2019; Elbasheir et al., 2024a; Wright et al., 2020), which may reduce statistical power and limited the ability to conduct meaningful subgroup or disorder-specific analyses. Several studies also noted the use of self-report measures, which may introduce recall bias or underreporting (Beatty Moody et al., 2019; Elbasheir et al., 2024b; Kral et al., 2024), and did not assess the psychological intensity of reported discrimination or real-time experiences of discrimination. Nevertheless, self-report measures remain a valuable approach for capturing experiences of racial discrimination, especially in contexts where experimental designs are not feasible. Although studies used different measures varying in time frame and items, all assess the same construct, so this is unlikely to be a major limitation.

Despite acknowledgment that experiences of racism should be examined through the lens of intersecting social identities and socioeconomic factors (Lewis & Grzanka, 2016), a major gap in the current literature is the lack of intersectional analyses, particularly for Black women, who experience unique forms of oppression (Fani et al., 2022a, b; Okeke et al., 2023). Additionally, many studies acknowledged that they did not investigate protective factors (e.g., coping strategies, resilience) that could moderate the effects of racial discrimination on brain health (Clark et al., 2018; Elbasheir et al., 2024b; Kral et al., 2024; Webb et al., 2022). However, early studies on specific phenomena often focus first on the direct effects of exposure, and these studies have made significant contributions to the field. Future research should examine how protective factors may moderate the effects of racial discrimination on brain outcomes. Furthermore, several studies were limited in their neuroimaging analysis, often focusing on a limited set of brain regions, such as the insula or amygdala, without exploring other areas potentially impacted by racial discrimination, such as those involved in stress regulation or immune system functioning (Fani et al., 2021; Kral et al., 2024).

Several studies reviewed here draw on data from the Grady Trauma Project, which were conducted within the Fani Lab (Elbasheir et al., 2024c; Fani et al., 2022b). The partial sample overlap may limit the independence of findings across studies. Future studies should explicitly document shared cohorts and emphasize replication in independent samples. Finally, all included studies were conducted in the United States, despite the presence of sizable Black populations in other parts of the world. For instance, more than 1.5 million people in Canada identify as Black, representing approximately 4.3% of the national population (Statistics Canada, 2022). In Europe, Black populations are similarly substantial. For example, France and the United Kingdom each have Black populations estimated between 3% and 5% of their total residents (Small, 2019). Despite the widespread presence of anti-Black racism and its well-documented psychosocial consequences in these regions, there has been a striking lack of scientific investment in examining its biological and epigenetic effects. The absence of neurobiological research on racism in these global contexts reflects a broader neglect that impedes the development of adapted clinical practice.

## Future directions

Future research on the neural mechanisms of racial discrimination should address several critical gaps identified in prior studies. First, longitudinal designs are needed to establish causal and temporal relationships between racial discrimination and neural, psychological, and physiological outcomes, overcoming the widespread reliance on cross-sectional data. Second, it is very important to apply intersectional approaches (e.g., gendered racism) to analyze the brain data. For example, researchers should consider gender, age, and geographic regions in their analyses to improve generalizability. Future studies would benefit from standardizing how covariates are handled or controlled. This should consider socioeconomic factors, such as education, income, employment, segregation, because these factors may co-occur with racial discrimination. That is, treating them as purely confounding variables may risk statistical overadjustment and conceptual misspecification. Therefore, it is essential to first articulate overarching conceptual models that clarify the role of socioeconomic covariates, and only then move toward more precise statistical specification. To address this, we also recommend using hierarchical or multilevel models that account for individual- and neighborhood-level factors to balance correlated exposures, longitudinal within-person designs that reduce time-invariant confounding, and sensitivity analyses that test the robustness of neural findings to different covariate specifications. Moreover, due to lack of studies on Indigenous people, and only one study on Latinx individuals, future research should conduct neuroimaging studies on the relation between racial discrimination and brain activities by disaggregating racialized groups in their analyses.

Third, studies should also incorporate larger sample sizes to increase statistical power and allow for disorder-specific and subgroup analyses. As well, it is important to report effect sizes to ensure the statistical power. Fourth, given the limitations of self-report measures, future research should utilize multimodal assessments, integrating neuroimaging, behavioral tasks, physiological markers (e.g., inflammation, glucocorticoids), and real-time experiences of racial discrimination. That is, it is also important to test real-time experiences of racial discrimination by presenting stimuli with racism contents during the fMRI scanning and asking participants to rate the intensity/valence/arousal level of such stimulus. We acknowledge there would be some ethical concerns regarding the real-time stimuli with racism content. To minimize this, we recommend using affective stimuli that show racist behaviours/discriminatory behaviours from already established movies/TV shows/video clips. In this line, our research team is currently conducting such experiment approved by the research ethics board.

Fifth, there is a need for more fine-grained analyses of brain regions, such as subregions of the insula or differential connectivity within salience and threat networks, to clarify mechanistic pathways. Sixth, research should assess and include protective factors such as coping strategies and resilience in their analyses, which may buffer adverse outcomes. Finally, future work should explore how structural racism and systemic inequities, beyond interpersonal racial discrimination, shape brain health across the lifespan and across generations.

## Conclusions

Resting-state neuroimaging studies provide compelling evidence that racial discrimination has significant effects on brain function and structure. Across developmental stages, from infancy to older adulthood, experiences of racial discrimination are associated with altered rsFC within brain regions responsible for emotion regulation, threat detection, memory, and self-awareness. Functional connectivity findings consistently reveal heightened connectivity in salience networks (e.g., amygdala-insula, amygdala-thalamus) and visual processing areas, which may reflect increased vigilance to social threats. Concurrently, reduced top-down regulation via decreased connectivity with prefrontal regions suggests potential vulnerabilities in emotion regulation processes. Structural imaging studies indicate decreased fractional anisotropy in white matter tracts such as the corpus callosum, cingulum bundle, and superior longitudinal fasciculus, which point to disruptions in neural integrity resulting from chronic race-related stress. Moreover, cortical thinning and volume reductions in regions like the anterior and posterior cingulate and hippocampus further suggest the neurobiological burden of racial discrimination. These changes have been linked to health outcomes, including accelerated biological aging, systemic inflammation, and increased risk for psychiatric symptoms and chronic disease.

In line with the CoRT model, these findings support the conceptualization of racial discrimination as a cumulative, multilevel stressor that becomes embedded in neurobiological systems across the lifespan. While consistent with the hypothesis proposed by Muscatell et al. (2022), that racial discrimination may involve networks related to affective processing, memory, and cognitive control, the evidence suggests a more complex and distributed pattern of neural impact that extends beyond these three networks. The involvement of widespread functional connectivity alterations, white matter integrity, cortical thickness, and volumetric changes indicates that racial discrimination should be understood as a multidimensional neurobiological process. These observations highlight the importance of developing a model that brings together functional and structural brain measures across the lifespan, helping us better understand how racial discrimination leaves lasting marks on the brain.

These findings emphasize that racial discrimination is not solely a psychosocial issue but a measurable biological stressor with profound implications for brain health. Ongoing and future research should continue to discover the causal mechanisms, developmental aspects of brain functions related to racial discrimination, and protective factors that may buffer against these effects to inform interventions regarding racial disparities in mental and physical health.

## Supplementary Information

Below is the link to the electronic supplementary material.

Supplementary file1 (DOCX 19 KB)

## Data Availability

Not applicable.
