# Supplementary material for: Racism and alterations in brain structure and function: A narrative review of emerging neuroimaging evidence in racialized individuals
Source: Cogn Affect Behav Neurosci. 2026 Jun 9;26(5):1962–82. doi: 10.3758/s13415-026-01461-x (PMC13615014; doi:10.3758/s13415-026-01461-x)
Supplement: Supplementary file 1 [file 13415_2026_1461_MOESM1_ESM.docx]

**Racism and the Brain: A Narrative Review of Emerging Neuroimaging Evidence in Racialized Individuals**

Seyed Mohammad Mahdi Moshirian Farahi ^1^

Jude Mary Cénat ^123^

^1^ School of Psychology, Faculty of Social Sciences, University of Ottawa, ON, Canada

^2^ Interdisciplinary Centre for Black Health, University of Ottawa, Ottawa, Ontario, Canada

^3^ University of Ottawa Research Chair on Black Health, Ottawa, Ontario, Canada

**Declarations**

**Funding**

This research project was funded by the Canadian Institute of Health Research (CIHR) with an Agency Reference Number of 200920.

**Conflicts of interest**

The authors have no potential conflicts of interest.

**Ethics approval**

Not applicable.

**Consent for publication**

All authors consent to publish this paper.

**Consent to participate**

Not applicable

**Availability of data and materials**

Not applicable.

**Code availability**

Not applicable.

**Acknowledgements**

We would like to thank Dr. Andra Smith for generously taking the time to read a draft of this article. Her thoughtful feedback and insights were greatly appreciated.

**Authors’ contribution**

**Dr. Moshirian Farahi:** Conceptualization, Investigation, Methodology, Visualization, Writing – original draft, Writing – review and editing, Funding acquisition. **Dr. Cénat:** Conceptualization, Writing – original draft, Writing – review and editing, Validation, Resources, Supervision.

*Corresponding authors*:

Seyed Mohammad Mahdi Moshirian Farahi, Ph.D.

School of Psychology, University of Ottawa, ON, Canada

[Smoshiri@uottawa.ca](mailto:Smoshiri@uottawa.ca)

AND

Jude Mary Cénat, Ph.D.

School of Psychology – University of Ottawa

136 Jean-Jacques-Lussier, 4017, Vanier Hall

Ottawa, Ontario, K1N 6N5

Phone: (613) 562-5800

Email: jcenat@uottawa.ca
